# Author Correction: Multiplexed imaging of high-density libraries of RNAs with MERFISH and expansion microscopy

**DOI:** 10.1038/s41598-018-24844-8

**Published:** 2018-04-19

**Authors:** Guiping Wang, Jeffrey R. Moffitt, Xiaowei Zhuang

**Affiliations:** Howard Hughes Medical Institute, Department of Chemistry and Chemical Biology, Department of Physics, Harvard University, Cambridge, MA 02138 USA

Correction to: *Scientific Reports* 10.1038/s41598-018-22297-7, published online 19 March 2018

This Article contains errors in the Methods section under subheading ‘Encoding probe staining’.

“Then 30 μL of ∼300 μM encoding probes and 3.3 μM of poly(dT) LNA anchor probe (a 20-nt sequence of alternating dT and thymidine-locked nucleic acid (dT+) with a 20-nt reverse complement of a readout sequence and a 5′-acrydite modification (Integrated DNA Technologies)) in encoding hybridization buffer was added to the surface of Parafilm (Bemis) and was covered with a cell-containing coverslip.”

should read:

“Then 30 μL of ∼50 μM encoding probes and 3.3 μM of poly(dT) LNA anchor probe (a 20-nt sequence of alternating dT and thymidine-locked nucleic acid (dT+) with a 20-nt reverse complement of a readout sequence and a 5′-acrydite modification (Integrated DNA Technologies)) in encoding hybridization buffer was added to the surface of Parafilm (Bemis) and was covered with a cell-containing coverslip.”

