# Supplementary material for: Multiplexed imaging of high-density libraries of RNAs with MERFISH and expansion microscopy
Source: Sci Rep. 2018 Mar 19;8:4847. doi: 10.1038/s41598-018-22297-7 (PMC5859009; doi:10.1038/s41598-018-22297-7)
Supplement: Supplementary file 1 — Supplementary Information [file 41598_2018_22297_MOESM1_ESM.pdf]

Supplementary Information for

Multiplexed imaging of high density libraries of RNAs with MERFISH and expansion microscopy

Guiping Wang[1], Jeffrey R. Moffitt[1], Xiaowei Zhuang[1*]
[1]Howard Hughes Medical Institute, Department of Chemistry and Chemical Biology, Department of Physics, Harvard University, Cambridge, MA 02138, USA

Supplementary Information includes:

Supplementary Figure 1

Supplementary Figure 2

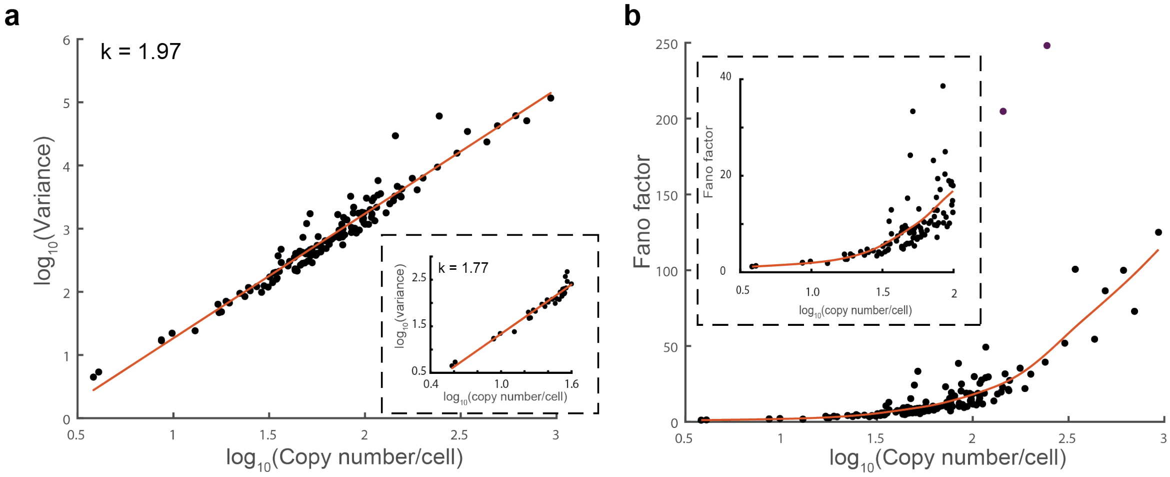

Supplementary Figure. 1. Cell-to-cell variation in the RNA copy number as a function of the RNA expression level for the ~130 genes measured in this work. **(a)** The variance in RNA copy number as a function of the RNA copy number for an expression range of $10^{0.5} – 10^{3}$ measured in this work. The red line is a power-law fit $\sigma_{RNA}^{2} \propto \mu_{RNA}^{k}$ with the scale exponent k = 1.97. Inset shows the data in the expression range of $10^{0.5} – 10^{1.6}$ copies per cell, and the power-law scaling coefficient in this range is slightly smaller, k = 1.77. This k value is similar to that derived previously from measuring the expression of one or two reporter genes incorporated into the chromosomes of T cells, which express RNAs in the same abundance range[25,26]. **(b)** The Fano factor, defined by the variance over the mean RNA copy numbers, as a function of the RNA expression level. The red line is the cubic spline fit of the data for a guide for the eye (Inset: zoom in to show the data in the expression range of $10^{0.5} – 10^{2}$ copies per cell. The trend observed is similar to previously published results[26,27]).

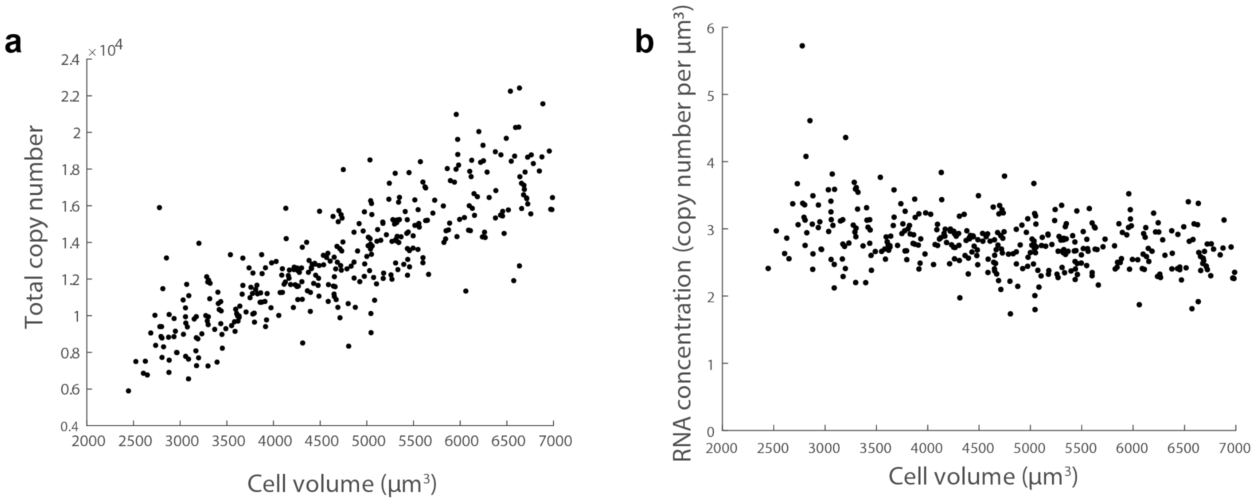

Supplementary Figure. 2. The total RNA copy number for the ~130 genes measured in each cell correlates linearly with the cell volume. **(a)** Scattered plot of the total RNA copy numbers of the 129 genes measured in single cells versus the cell volume. Cell volume was calculated by computing the volume of a 3D convex hull covering all RNAs in a cell. **(b)** Scatter plot of the RNA concentration, defined as total copy number per cell divided by the cell volume, versus the cell volume.
